# A closer look at the relationship among accelerometer-based physical activity metrics: ICAD pooled data

**DOI:** 10.1186/s12966-019-0801-x

**Published:** 2019-04-29

**Authors:** Soyang Kwon, Lars Bo Andersen, Anders Grøntved, Elin Kolle, Greet Cardon, Rachel Davey, Susi Kriemler, Kate Northstone, Angie S. Page, Jardena J. Puder, John J. Reilly, Luis B. Sardinha, Esther M. F. van Sluijs, Kathleen F. Janz

**Affiliations:** 1 Ann & Robert H. Lurie Children’s Hospital of Chicago Stanley Manne Children’s Research Institute, 225 E Chicago Ave, Box 157, Chicago, IL 60611 USA; 2 Faculty of Education, Arts and Sport, Western Norway University of Applied Sciences, Sogndal, Norway; 3 Department of Sports Science and Clinical Biomechanics, University of Southern Denmark, Odense, Denmark; 4 Norway, Norwegian School of Sport Science, Oslo, Norway; 5 Department of Movement and Sports Sciences, Ghent University, 9000 Ghent, Belgium; 6 Centre for Research & Action in Public Health Health Research Institute, University of Canberra, Canberra, Australia; 7 Epidemiology, Biostatistics and Prevention Institute, University of Zürich, Zürich, Switzerland; 8 Bristol Medical School, University of Bristol, Bristol, UK; 9 Centre for Exercise, Nutrition and Health Sciences, University of Bristol, Bristol, UK; 10 Obstetric service, Lausanne University Hospital, Lausanne, Switzerland; 11 Physical Activity for Health Group, School of Psychological Sciences and Health, University of Strathclyde, Glasgow, UK; 12 Exercise and Health Laboratory, CIPER, Faculdade de Motricidade Humana, Universidade de Lisboa, Cruz-Quebrada, Portugal; 13 Centre for Diet and Activity Research (CEDAR) & MRC Epidemiology Unit, University of Cambridge, Cambridge, UK; 14 Department of Health and Human Physiology, University of Iowa, Iowa City, IA USA

**Keywords:** ICAD, Children, Adolescents, ActiGraph, Total activity counts, Sedentary, Physical activity measurement

## Abstract

**Background:**

Accelerometers are widely used to assess child physical activity (PA) levels. Using the accelerometer data, several PA metrics can be estimated. Knowledge about the relationships between these different metrics can improve our understanding of children’s PA behavioral patterns. It also has significant implications for comparing PA metrics across studies and fitting a statistical model to examine their health effects. The aim of this study was to examine the relationships among the metrics derived from accelerometers in children.

**Methods:**

Accelerometer data from 24,316 children aged 5 to 18 years were extracted from the International Children’s Accelerometer Database (ICAD) 2.0. Correlation coefficients between wear time, sedentary behavior (SB), light-intensity PA (LPA), moderate-intensity PA (MPA), vigorous-intensity PA (VPA), moderate- and vigorous-intensity PA (MVPA), and total activity counts (TAC) were calculated.

**Results:**

TAC was approximately 22X10^3^ counts higher (*p* < 0.01) with longer wear time (13 to 18 h/day) as compared to shorter wear time (8 to < 13 h/day), while MVPA was similar across the wear time categories. MVPA was very highly correlated with TAC (*r* = .91; 99% CI = .91 to .91). Wear time-adjusted correlation between SB and LPA was also very high (*r* = −.96; 99% CI = -.96, − 95). VPA was moderately correlated with MPA (*r* = .58; 99% CI = .57, .59).

**Conclusions:**

TAC is mostly explained by MVPA, while it could be more dependent on wear time, compared to MVPA. MVPA appears to be comparable across different wear durations and studies when wear time is ≥8 h/day. Due to the moderate to high correlation between some PA metrics, potential collinearity should be addressed when including multiple PA metrics together in statistical modeling.

**Electronic supplementary material:**

The online version of this article (10.1186/s12966-019-0801-x) contains supplementary material, which is available to authorized users.

## Background

Accelerometers have become a widely used tool to assess physical activity (PA) levels among children. Using accelerometer data, several important public health-related PA metrics, including time spent in sedentary behavior (SB), light-intensity PA (LPA), moderate-intensity PA (MPA), vigorous-intensity PA (VPA), and moderate- to vigorous-intensity PA (MVPA), can be estimated. Daily accumulated accelerometer counts (total activity counts; TAC) has also been suggested as a metric of total PA volume [1–3]. TAC can be conceptualized as a proxy of the total PA volume that encompasses the frequency, intensity, and duration of activity bouts [2]. Despite the conceptual distinctions between TAC and PA intensity metrics, there is a knowledge gap in how TAC is related to time spent in individual PA intensity categories and if wear time affects TAC differently than the individual PA intensity categories. For example, it is probable that TAC is mostly explained by accelerometer counts collected during MVPA. Also, TAC could be more dependent on wear time than MVPA.

Accelerometer wear time is a key variable that could significantly impact the accelerometer-derived PA metrics. Although researchers apply an accelerometer data inclusion criterion, for example, at least 8 wear hours per day, to estimate a PA level that can reflect all-day PA, still there are wide variations in wear time from 8 h to 24 h per day. Thus, researchers often implement additional approaches (e.g., adjust for wear time in statistical modeling [4]) to standardize/adjust PA metrics within a study population. However, a wear time-dependent PA metric has a limitation when comparing the results across studies that have different wear times. That metric may require additional processing to standardize the metric for a comparison. Given that children spend ≥90% of waking time in lower intensity activities such as SB and LPA [5], longer wear time during waking time would capture more SB and LPA. Therefore, it can be hypothesized that TAC, which includes SB and LPA, are more dependent on wear time than MVPA.

As diverse activity metrics can now be calculated using accelerometer data beyond MVPA, which was traditionally the sole focus of PA research when using PA questionnaires, more recent studies have examined the health effects of these diverse accelerometer data-derived metrics. Some, but not all studies [6–8], report that independent of MVPA time, sedentary time is associated with poor health outcomes [9, 10], while LPA time is associated with favorable health outcomes [5, 10, 11]. From this perspective, determining which PA behaviors/metrics are more important has become a great public health concern [6, 8, 12, 13]. Although each of the PA intensity metrics conceptually represents a distinct behavior with potentially unique determinants and ascertaining their effects has significant clinical implications, quantifying their independent effects is a challenge. First, because the sum of the accelerometer data-derived metrics (i.e., sum of MVPA, LPA, and SB) results in a finite total accelerometer wear time and time spent in one activity level necessarily displaces time spent in at least another activity level [13], these metrics are inherently co-dependent. Second, because SB, LPA, MPA, and VPA behaviors/metrics are often correlated with each other in empirical data [14, 15], this could cause a collinearity problem in a multivariable regression model that includes multiple PA metrics together as predictors. Even when using an alternative approach that fits a regression model for each of the PA intensity metrics individually [12], the results have the limitation of not accounting for the effects of other PA intensity metrics. Knowledge about the relationships between the accelerometer data-derived PA metrics will help to improve our understanding of PA behavior patterns among children and provide more coherent methodological recommendations in the use of PA metrics.

For this study, we had the following hypotheses: (1) TAC is highly correlated with MVPA and mostly explained by MVPA, (2) TAC is more dependent on wear time than MVPA, and (3) some PA intensity metrics are strongly correlated with each other. To test these hypotheses, this study examined the relationships among accelerometer-derived PA metrics in children. These relationships were further examined by individual characteristics, such as sex, age, mother’s education level, and obesity status, which have been reported to be associated with PA levels in previous studies [16–18]. The stratified analyses were conducted to ensure the correlations among PA intensity metrics are similar and therefore not confounded by the individual characteristics. This study also examined a correlation between wear time and PA metrics.

## Methods

### Study participants

This study used data from the International Children’s Accelerometer Database (ICAD) 2.0. ICAD, an international pooled database for 20 studies that collected ActiGraph accelerometer data among children. Of the 20 studies, the CHAMPS-US study that conducted three waves of accelerometer assessment within two weeks was excluded from this study. Of the 19 included studies, 12 were conducted in European countries, four in the United States, two in Australia, and one in Brazil. Six were cross-sectional studies, 10 were cohort studies, and four were intervention studies. ICAD obtained and processed raw ActiGraph accelerometry data files from the partner studies. Details on the design and methods of ICAD are described elsewhere [19, 20]. The ICAD 2.0 included 51,434 accelerometer assessments from the 19 studies. For the current analyses, we excluded the post-intervention data of the four intervention studies (*n* = 9687). We further excluded data of participants whose age value was higher than 18 (*n* = 2), lower than 5 (*n* = 212), or missing (*n* = 949). Because the Magic study only included participants aged 3 and 4 years old, all of the Magic participants were excluded based on the age eligibility criterion. Next, we excluded the spurious ActiGraph data (*n* = 520) as described in Sherar et al. [19] After additionally removing 4435 assessments with < 3 valid accelerometer wear days (valid wear day was defined as ≥480 min of valid wear time between 6:00 AM and midnight (12:00 AM) and total activity counts < 1000,000 counts), 35,629 assessments from 24,316 children aged 5 to 18 years were identified. Of those, only the first valid assessment data (*n* = 24,316) were included in the current data analysis.

### Data elements

The ICAD raw accelerometer data were processed using specifically developed and commercially available software (KineSoft, Saskatchewan, Canada). This process has been described in detail elsewhere [12]. Briefly, accelerometry data files were (re)integrated to 60-s epochs. Non-wear time was defined as consecutive zero counts for ≥60 min, allowing for two minutes of non-zero interruptions [21]. For the present analysis, we only used accelerometer data collected between 6:00 AM and midnight to (partially) exclude the data collected during nighttime sleep. Accelerometer-derived metrics analyzed included wear time (minutes/day) and TAC (accumulated vertical axis accelerometer counts/day), as well as SB (accumulated time in minutes/day with 0 to 100 accelerometer counts/minute), LPA (accumulated time in minutes/day with 101 to 2295 accelerometer counts/minute), MPA (accumulated time in minutes/day with 2296 and 4011 cpm), VPA (accumulated time in minutes/day with ≥4012 cpm), and MVPA (accumulated time in minutes/day with ≥2296 cpm), based on Evenson’s cut-points [22, 23].

ICAD harmonized mother’s education level data into three categories: (1) up to and including completion of compulsory education, (2) some post-compulsory education or vocational training, and (3) completed undergraduate or postgraduate education. The detailed information for the harmonization process can be found at http://www.mrc-epid.cam.ac.uk/research/studies/icad/data-harmonisation/. Five of the 18 studies did not collect/provide mother’s education data. An obesity variable (obese vs. non-obese) was created based on the age- and sex-specific body mass index (BMI) cut-points that correspond to BMI of 30 kg/m^2^ for age 18 years [24].

### Statistical analysis

All analyses were conducted in SAS 9.4. (Cary, NC). Before pooled data analyses, the heterogeneity between the studies was examined by comparing the correlation coefficients between accelerometer-derived PA metrics for each study (Additional file 1: Table S1). The results were considered consistent across the studies, and therefore we proceeded to pooled analyses. Among the 18 studies, the median sample size was 1103. Ten studies had a sample size larger than 1000. Studies with more than 1000 participants were weighted so that the weighted sample size became 1000. We used this approach, rather than simple inverse variance weighting, to allow for more contribution of the larger studies, and, at the same time, to avoid domination by a very large study (i.e., a study with 6514 participants).

Descriptive analysis was conducted for accelerometer-derived metrics. Because the range of PA metrics varied greatly by age, we calculated correlation coefficients of wear time and PA metrics by age group (5 to 9, 10 to 12, 13 to 15, and 16 to 18 years). To test the first hypothesis, we conducted linear regression analysis for TAC predicted by MVPA (Model 1), and then further by wear time (Model 2) or by LPA residuals (Model 3). Standardized LPA residuals were created by regressing MVPA on LPA, to account for the correlation between LPA and MVPA. Correlation analyses between PA metrics were conducted, separately by age group, sex, mother’s education, obesity, type of assessment day (weekday and weekend day), and wear time (8 to < 13 and 13–18 h/day). We further calculated the proportion of VPA within MVPA (VPA minutes ÷ MVPA minutes × 100) for each of the MVPA level categories (< 20, 20 to < 40, 40 to < 60, 60 to < 80, and ≥ 80 min/day) to examine whether more active children were proportionally more involved in VPA. A significance level was set at 0.01, and 99% confidence intervals (CI) were calculated.

## Results

Among 24,316 participants, 10.7% had weekday data only, and 7.3% were categorized as obese. The median number of valid wear days was 6 days (interquartile range of 4 to 7 days).

Means and 99% CIs of accelerometer-derived metrics are presented in Table 1. Mean SB, LPA, and TAC were significantly higher among children who wore a monitor for 13 to 16 h per day, compared to those who did for 8 to < 13 h per day. However, MPA and VPA were similar across the wear time categories. Among boys, while MVPA level was maintained until age 10–12 years and then declined, TAC declined over time, being the highest at age 5–9 years and the lowest at age 16–18 years.

**Table 1:** Means and 99% confidence intervals of accelerometer-derived metrics

	Unweighted sample	Wear minutes	Sedentary minutes	LPA minutes	MPA minutes	VPA minutes	TAC × 1000
	n			Mean (99% CI)			
All	24,316	776 (774, 777)	382 (380, 383)	346 (345, 347)	35 (34, 35)	13 (13, 13)	417 (415, 420)
Age and sex*							
5–9 yr. boy	2558	758 (754, 763)	303 (298, 307)	395 (392, 399)	45 (44, 46)	16 (15, 16)	518 (510, 525)
5–9 yr. girl	2740	755 (751, 759)	306 (302, 310)	400 (397, 404)	37 (36, 37)	12 (11, 12)	467 (460, 473)
10–12 yr. boy	4547	768 (765, 771)	348 (345, 351)	358 (355, 360)	45 (44, 46)	17 (17, 18)	488 (483, 493)
10–12 yr. girl	6701	769 (767, 772)	375 (373, 378)	354 (352, 356)	30 (30, 30)	11 (10, 11)	392 (388, 395)
13–15 yr. boy	2810	804 (799, 808)	445 (440, 450)	308 (304, 312)	33 (33, 33)	17 (17, 18)	399 (392, 406)
13–15 yr. girl	3160	799 (795, 803)	472 (468, 476)	292 (288, 295)	26 (25, 26)	10 (10, 11)	317 (311, 322)
16–18 yr. boy	872	790 (781, 798)	459 (451, 468)	288 (281, 296)	27 (26, 29)	15 (14, 16)	353 (341, 365)
16–18 yr. girl	922	788 (781, 796)	481 (474, 489)	279 (273, 286)	20 (19, 21)	7 (7, 8)	274 (265, 283)
Mother’s education level**							
<=compulsory education	6192	767 (764, 769)	356 (353, 359)	361 (359, 364)	36 (35, 36)	14 (13, 14)	437 (432, 442)
Some post-compulsory education or vocational	4766	777 (773, 780)	368 (365, 372)	360 (357, 362)	35 (35, 36)	13 (13, 14)	431 (426, 436)
> = completed undergraduate	4090	778 (775, 781)	375 (371, 379)	354 (351, 357)	35 (34, 36)	14 (14, 15)	431 (425, 437)
Obesity status***							
Non-obese	21,728	777 (775, 778)	381 (379, 382)	347 (346, 349)	35 (35, 35)	14 (14, 14)	422 (419, 425)
Obese	1767	767 (762, 772)	392 (385, 398)	338 (334, 343)	28 (27, 29)	9 (8, 9)	363 (356, 371)
Type of day							
Weekday	24,316	795 (793, 796)	394 (392, 396)	349 (348, 351)	37 (37, 37)	14 (14, 15)	433 (430, 435)
Weekend	21,698	732 (730, 734)	352 (350, 354)	341 (339, 342)	29 (28, 29)	11 (10, 11)	382 (379, 385)
Wear time							
8- < 13 h/day	12,817	714 (713, 716)	337 (335, 339)	330 (329, 332)	34 (34, 34)	13 (12, 13)	407 (404, 410)
13–18 h/day	11,499	844 (843, 846)	431 (429, 434)	364 (362, 366)	35 (35, 36)	14 (14, 14)	429 (425, 432)

*n for missing = 6

**n for missing = 9268

***n for missing = 821

CI, confidence interval; LPA, light-intensity physical activity; MPA, moderate-intensity physical activity; TAC, total activity counts; VPA, vigorous-intensity physical activity

### Correlation coefficients between wear time and PA metrics

We describe the correlation levels based on Mukaka’s suggestion: ‘negligible’ for *r* = .0 to .3; ‘low’ or “weak” for *r* = .3 to .5; ‘moderate’ for *r* = .5 to .7; ‘high’ for *r* = .7 to .9; and ‘very high’ for *r* = .9 to 1.0 [25]. As presented in Table 2, wear time was correlated with SB and LPA at a low to moderate level. The correlations of wear time with TAC and MVPA were both at a negligible level, although the correlation coefficients of wear time with TAC were significantly higher than those with MVPA.

**Table 2:** Correlation coefficients of wear time with physical activity metrics

	SB	LPA	MPA	VPA	MVPA	TAC
	*r* (99% CI)					
All	.57 (.56, .58)	.30 (.29, .32)	.06 (.05, .07)	.05 (.04, .07)	.06 (.05, .08)	.12 (.10, .13)
Age						
5–9 years	.53 (.52, .55)	.44 (.42, .46)	.12 (.09, .14)	.08 (.05, .11)	.11 (.09, .14)	.20 (.17, .22)
10–12 years	.57 (.56, .59)	.49 (.47, .50)	.10 (.08, .12)	.06 (.04, .08)	.09 (.07, .11)	.19 (.18, .21)
13–15 years	.57 (.56, .59)	.44 (.42, .46)	.13 (.10, .15)	.02 (−.01, .05)	.09 (.07, .12)	.20 (.17, .22)
16–18 years	.59 (.56, .62)	.42 (.38, .46)	.19 (.15, .23)	.12 (.07, .22)	.18 (.13, .22)	.28 (.24, .32)

CI, confidence interval; LPA, light-intensity physical activity; MPA, moderate-intensity physical activity; MVPA, moderate-and vigorous-intensity physical activity; SB, sedentary behavior; TAC, total activity counts; VPA, vigorous- intensity physical activity

### Correlation coefficients between PA metrics

**Fig. 1:**
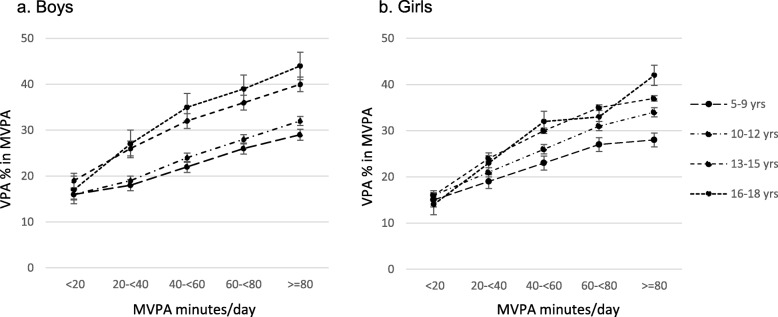
The proportion of vigorous-intensity physical activity (VPA) minutes within moderate- and vigorous-intensity physical activity (MVPA) minutes. Note. Error bar indicates 99% confidence interval

As shown in Table 3, although the correlation between SB and LPA was overall moderate (*r* = −.58), when it was examined by wear time, it became high (*r* = −.77 and − .78 by wear time categories) or very high (wear time-adjusted *r* = −.96). The overall correlation coefficient between MVPA and LPA was .32. Regardless of age, sex, mother’s education, obesity status, type of day assessed, and length of wear time, MVPA was correlated with TAC at a very high level (overall *r* = .91). The correlation coefficient between MPA and VPA was overall .58. In further exploration, we found a trend that the proportion of VPA within MVPA increased as MVPA increased (Fig. 1), indicating that children who are more active engage in proportionally more VPA.

**Table 3:** Correlation coefficients between accelerometer-derived physical activity metrics

	SB & LPA	MVPA & SB	MVPA & LPA	MVPA & TAC	MPA & VPA
			*r* (99% CI)		
All	−.58 (−.59, −.57)	−.43 (−.44, −.42)	.32 (.30, .33)	.91 (.91, .91)	.58 (.58, .59)
Age					
5–9 years	−.48 (−.50, −.46)	.42 (−.44, −.39)	.28 (.25, .30)	.92 (.92, .92)	.65 (.63, .67)
10–12 years	−.39 (−.40, −.37)	−.42 (−.43, −.40)	.26 (.24, .28)	.92 (.92, .93)	.65 (.63, .66)
13–15 years	−.45 (−.47, −.43)	−.38 (−.40, −.35)	.25 (.23, .28)	.92 (.92, .92)	.52 (.51, .54)
16–18 years	−.45 (−.49, −.41)	−.22 (−.26, −.18)	.18 (.14, .23)	.91 (.90, .91)	.58 (.54, .61)
Sex					
Boy	−.57 (−.58, −.55)	−.41 (−.43, −.39)	.30 (.28, .32)	.91 (.91, .92)	.51 (.50, .53)
Girl	−.61 (−.62, −.60)	−.40 (−.42, −.30)	.34 (.32, .35)	.89 (.89, .89)	.59 (.58, .60)
Mother’s education level					
<=Compulsory education	−.52 (−.54, −.50)	−.46 (−.48, −.44)	.32 (.30, .34)	.92 (.92, .93)	.66 (.64, .67)
Some post-compulsory education or vocational	−.53 (−.55, −.51)	−.43 (−.45, −.40)	.35 (.32, 37)	.92 (.92, .92)	.63 (.61, .64)
> = Completed undergraduate	−.61 (−.63, −.59)	−.49 (−.51, −.47)	.36 (.33, .39)	.93 (.92, .93)	.64 (.62, .66)
Obesity status					
Non-obese	−.58 (−.59, −.58)	−.43 (−.44, −.42)	.32 (.30, .33)	.91 (.91, .92)	.58 (.57, .59)
Obese	−.55 (−.58, −.52)	−.42 (−.45, −.38)	.30 (.26, .35)	.89 (.88, .90)	.59 (.55, .62)
Type of day					
Weekday	−.56 (−.57, −.55)	−.40 (−.41, −.38)	.30 (.29, .31)	.91 (.91, .91)	.56 (.55, .57)
Weekend	−.45 (−.46, −.44)	−.39 (−.40, −.38)	.32 (.31, .33)	.91 (.90, .91)	.58 (.57, .59)
Wear time					
8- < 13 h/day	−.77 (−.78, −.76)	−.52 (−.53, −.50)	.32 (.30, .33)	.91 (.91, .92)	.61 (.60, .62)
13–18 h/day	−.78 (−.79, −.78)	−.49 (−.51, −.48)	.31 (.29, .33)	.91 (.91, .91)	.56 (.55, .57)
Wear time (continuous variable)-adjusted	−.96 (−.96, −.95)	−56 (−.57, −.55)	.31 (.30, .31)	.91 (91, .91)	.58 (.57, .59)

CI, confidence interval; LPA, light-intensity physical activity; MPA, moderate-intensity physical activity; MVPA, moderate-and vigorous-intensity physical activity; SB, sedentary behavior; TAC, total activity counts; VPA, vigorous-intensity physical activity

### Linear regression models for TAC predicted by MVPA

As presented in Table 4, MVPA solely explained 83% of variation in TAC. Adding the wear time variable improved the R^2^ only 0.4%, although one-minute longer wear time was significantly associated with 100-count higher TAC. Adding the LPA variable improved the R^2^ by 12% (R^2^ = 0.95).

**Table 4:** Linear regression models to predict daily total activity counts by time spent in moderate- and vigorous-intensity physical activity and wear time

	Model 1	Model 2	Model 3
*R* ^*2*^	0.832	0.836	0.949
		Estimate (99% CI)	
Intercept	134,711 (162,594, 166,828)	87,797 (78,716, 96,877)	164,769 (163,602, 165,936)
MVPA, minutes/day	5279 (5239,5318)	5257 (5218, 5296)	5280 (5258, 5302)
Wear time, minutes/day	–	100 (89, 112)	–
LPA, transformed minutes/day*	–	–	664 (657, 672)

*Standardized residuals of LPA were created by regressing MVPA on LPA. The standardized residual variable was used for regression modeling and the parameter estimate was transformed to express it in LPA minutes/day

CI, confidence interval; MVPA, moderate-and vigorous-intensity physical activity

## Discussion

This study examined the relationships among accelerometer-derived PA metrics, using data from the largest available child accelerometer database. We found that TAC was mostly (83%) explained by MVPA. We also found that TAC, compared to MVPA, is more dependent on wear time. SB, LPA, and TAC were higher with longer wear time, although MPA, VPA, and MVPA were similar across different wear durations when daily wear time was 8 h or higher. SB was negatively correlated with LPA at a high level. Our results also indicate that children who are more active engage in proportionally more VPA.

Accelerometer data collected in the field are often unable to provide accelerometer data during the *whole* waking hours (complete data), due to later put-on in the morning or earlier take-off before sleep, for example. Therefore, it would be ideal to use a PA measure that is least affected by data completeness, so that the measure can be directly comparable across studies. If a PA metric is highly variable depending on the level of data completeness (or wear time) and therefore requires complete data to avoid the bias associated with incomplete data, this would decrease the data usability in epidemiologic studies. This would also potentially eliminate the ability to compare the metrics derived from different wear times across studies. The present study found that SB and LPA were correlated with wear time at a low to moderate level. The present study also found that, despite the high collinearity between MVPA and TAC and a negligible level of correlation between wear time and TAC, the correlation of wear time with TAC was still higher than the correlation with MPA, VPA, or MVPA, and mean TAC was significantly higher with longer wear time. This implies that TAC could be more affected by data completeness than MPA, VPA, or MVPA. Altogether, these suggest that if most of the data during waking hours are captured (e.g., ≥8 h/day), the bias associated with incomplete data could be minimal for VPA, MPA, and MVPA metrics, but not for SB, LPA, or TAC. This knowledge should increase confidence in study results that examine MPA, VPA, and MVPA, despite inevitable compliance issues. It should also increase confidence that MPA, VPA, and MVPA metrics can be compared across different studies that are likely to have different wear times or data completeness.

Bassett and colleagues [1] have argued that TAC may be a better metric than PA intensity metrics because it incorporates the full continuum of PA intensities. Although this is conceptually true, LPA may not be well reflected in TAC in empirical data. Rather, TAC may be mostly explained by MVPA. In fact, this study revealed that TAC is 83% explained by MVPA, while it is only 12% explained by LPA. Our findings emphasize the need for a closer examination of the link between accelerometer counts and PA intensity to better assess whether TAC can be a meaningfully different measure from MVPA in empirical data. For example, despite two decades of accelerometer-measured PA studies, research has not yet closely examined whether an increase in accelerometer counts is proportional to an increase in PA intensity (e.g., expressed in metabolic equivalents). Also, it is an interesting finding that among boys, while MVPA was maintained until preadolescence and then declined, TAC declined over time from age 5 to 18 years. This result could indicate that children maintain MVPA level, but reduce LPA level during preadolescence. However, this result should be further validated to determine whether it is a reflection of true behavioral change during preadolescence or measurement error for MVPA or TAC. Further, as mentioned above, TAC has a stronger dependency on monitor wear time as compared to MVPA. Altogether, we suggest that the added value of the TAC metric in PA research be further discussed by the research community.

This study reveals an interesting relationship between MVPA and VPA. Children with higher MVPA levels engaged in proportionally more VPA. For example, children who engaged in MVPA for 40 to 60 min daily engaged in VPA for 13 min (26% of total MVPA) on average, while children who engaged in MVPA for ≥80 min daily engaged in VPA for 32 min (33% of total MVPA), on average. This tendency was much clearer among older children than younger children. For example, among those with ≥80 min of MVPA, the VPA proportion was 29% for the youngest age group (5 to 9 years), while it was 44% for the oldest age group (16 to18 years). However, the tendency was not different between boys and girls. Although girls on average had lower MVPA, the proportion of VPA at a given MVPA level was similar between boys and girls. Based on these findings, we presume that sports participation, particularly vigorous-intensity sports participation among older children, largely contributes to children’s active lifestyle. However, the interpretation of our results by age groups requires caution since higher VPA proportion among older children could be partly due to use of the same cut-point for VPA across all ages. Our findings also suggest that children who are active are likely to receive the health benefits of VPA, presumably due to their high engagement in VPA.

Examining the effects of the PA metrics is important to establish an evidence base for the health benefits of PA. It is a commonly used statistical approach to include two or more PA intensity metrics in one statistical model to examine their health effects. However, our findings of the substantial correlations between some PA metrics suggest that this approach might violate the non-collinearity assumption required for regression models. To address the collinearity issue, several alternative approaches can be considered. One could divide the study population into subgroups based on PA patterns, for example using cluster analysis, and then compare the health outcomes of those groups. The use of residuals could be another statistical approach. In addition, considering the inherent co-dependency of PA intensity metrics [13], studies that examine the allocation of the 24-h period to the full spectrum of activities, including sleep, SB, LPA, MPA, and VPA, could utilize the compositional data analysis method [5, 26–29].

Several limitations of the study should be acknowledged. First, despite our attempt to (partially) exclude sleep time data by using the data collected only from 6 AM to midnight, this time frame could still include some data during sleep time, which could have biased our results, particularly SB estimation. However, given that only two of the 18 studies (3% of the sample) used a 24-h accelerometer protocol and the remaining 16 studies used a waking-hour protocol, we expect that the bias would be minimal (Additional file 1: Table S2). Second, although we believe that TAC should be defined as accumulated counts during LPA, MPA, and VPA, but not during SB [3, 8], we used the TAC variable that was defined as accumulated counts during SB through VPA, because the ICAD 2.0 dataset did not contain the TAC variable that only included LPA to VPA. Lastly, with the selection of different cut-points, non-wear criteria, or epoch length, the correlation coefficients that we observed could change. However, we selected to use the most widely accepted accelerometer data reduction methodologies.

## Conclusions

This study provides insights to the pediatric PA research community regarding the selection of accelerometer-based PA metrics and to the investigation of the health effects of PA. This study found that TAC is mostly explained by MVPA, while it could be more dependent on wear time, compared to MVPA. MVPA appears to be comparable across different wear durations and studies when wear time is ≥8 h/day. Because of moderate to high correlations between some PA intensity metrics, potential collinearity should be addressed when including them together in statistical modeling to examine their health effects.

## Additional file

Additional file 1: **Table S1.** Correlation coefficients between accelerometer-derived physical activity metrics by studies. **Table S2.** Correlation coefficients between accelerometer-derived metrics for the 16 studies with the walking-hour accelerometer protocol. (DOCX 26 kb)

## Abbreviations

BMI: Body mass index
CI: Confidence interval
ICAD: International Children’s Accelerometer Database
LPA: Light-intensity physical activity
MPA: Moderate-intensity physical activity
MVPA: Moderate- and vigorous-intensity physical activity
PA: Physical activity
SB: Sedentary behavior
TAC: Total activity counts
VPA: Vigorous-intensity physical activity
