# Correction to: A closer look at the relationship among accelerometer-based physical activity metrics: ICAD pooled data

**DOI:** 10.1186/s12966-019-0828-z

**Published:** 2019-08-08

**Authors:** Soyang Kwon, Lars Bo Andersen, Anders Grøntved, Elin Kolle, Greet Cardon, Rachel Davey, Susi Kriemler, Kate Northstone, Angie S. Page, Jardena J. Puder, John J. Reilly, Luis B. Sardinha, Esther M. F. van Sluijs, Kathleen F. Janz, On behalf of the ICAD Collaborators

**Affiliations:** 1 Ann & Robert H. Lurie Children’s Hospital of Chicago Stanley Manne Children’s Research Institute, 225 E Chicago Ave, Box 157, Chicago, IL 60611 USA; 2 Faculty of Education, Arts and Sport, Western Norway University of Applied Sciences, Sogndal, Norway; 3 Department of Sports Science and Clinical Biomechanics, University of Southern Denmark, Odense, Denmark; 4 Norway, Norwegian School of Sport Science, Oslo, Norway; 5 Department of Movement and Sports Sciences, Ghent University, 9000 Ghent, Belgium; 6 Centre for Research & Action in Public Health Health Research Institute, University of Canberra, Canberra, Australia; 7 Epidemiology, Biostatistics and Prevention Institute, University of Zürich, Zürich, Switzerland; 8 Bristol Medical School, University of Bristol, Bristol, UK; 9 Centre for Exercise, Nutrition and Health Sciences, University of Bristol, Bristol, UK; 10 Obstetric Service, Lausanne University Hospital, Lausanne, Switzerland; 11 Physical Activity for Health Group, School of Psychological Sciences and Health, University of Strathclyde, Glasgow, UK; 12 Exercise and Health Laboratory, CIPER, Faculdade de Motricidade Humana, Universidade de Lisboa, Cruz-Quebrada, Portugal; 13 Centre for Diet and Activity Research (CEDAR) & MRC Epidemiology Unit, University of Cambridge, Cambridge, UK; 14 Department of Health and Human Physiology, University of Iowa, Iowa City, IA USA

**Correction to: Int J Behav Nutr Phys Act (2019) 16:40**

**https://doi.org/10.1186/s12966-019-0801-x**

Following publication of the original article [1], the author reported that the name of the collaborator group was missing from the author group.

Author group in published article:

Soyang Kwon1*, Lars Bo Andersen2, Anders Grøntved3, Elin Kolle4, Greet Cardon5, Rachel Davey6, Susi Kriemler7, Kate Northstone8, Angie S. Page9, Jardena J. Puder10, John J. Reilly11, Luis B. Sardinha12, Esther M. F. van Sluijs13 and Kathleen F. Janz14.

Revised author group:

Soyang Kwon1*, Lars Bo Andersen2, Anders Grøntved3, Elin Kolle4, Greet Cardon5, Rachel Davey6, Susi Kriemler7, Kate Northstone8, Angie S. Page9, Jardena J. Puder10, John J. Reilly11, Luis B. Sardinha12, Esther M. F. van Sluijs13 and Kathleen F. Janz14 On behalf of the ICAD Collaborators.
