# Supplementary material for: A closer look at the relationship among accelerometer-based physical activity metrics: ICAD pooled data
Source: Int J Behav Nutr Phys Act. 2019 Apr 29;16:40. doi: 10.1186/s12966-019-0801-x (PMC6489360; doi:10.1186/s12966-019-0801-x)
Supplement: Supplementary file 1 — Table S1. Correlation coefficients between accelerometer-derived physical activity metrics by studies. Table S2. Correlation coefficients between accelerometer-derived metrics for the 16 studies with the walking-hour accelerometer protocol. (DOCX 26 kb) [file 12966_2019_801_MOESM1_ESM.docx]

Additional file 1: Table S1 Correlation coefficients between accelerometer-derived physical activity metrics by studies

| Study name | Sample size | Age  in years | Wear minutes | SB & LPA | MVPA & SB | MVPA & LPA | MVPA & TAC | MPA & VPA |
| --- | --- | --- | --- | --- | --- | --- | --- | --- |
|  | n | Median (min, max) | Mean | *r* | *r* | *r* | *r* | *r* |
| SPEEDY | 1,959 | 10 (9, 15) | 740 | -.39 | -.34 | .19 | .87 | .52 |
| PEACH | 1,199 | 11 (9, 16) | 746 | -.34 | -.36 | .17 | .89 | .56 |
| ALSPAC | 6,514 | 13 (10, 17) | 775 | -.69 | -.42 | .32 | .93 | .54 |
| Ballabeina | 196 | 5 (5, 6) | 725 | -.21 | -.27 | .21 | .91 | .63 |
| CLAN | 1,135 | 11 (5, 17) | 761 | -.58 | -.45 | .20 | .90 | .61 |
| COSCIS | 546 | 6 (5, 8) | 740 | -.34 | -.32 | .11 | .92 | .66 |
| EYHS Denmark | 1,616 | 12 (8, 18) | 791 | -.82 | -.54 | .48 | .93 | .67 |
| EYHS Estonia | 615 | 12 (7, 17) | 799 | -.75 | -.50 | .26 | .92 | .36 |
| EYHS Norway | 347 | 9 (8, 10) | 782 | -.80 | -.72 | .56 | .95 | .70 |
| EYHS Portugal | 1,070 | 11 (8, 17) | 784 | -.70 | -.49 | .33 | .92 | .64 |
| HEAPS | 1,306 | 10 (5, 15) | 727 | -.40 | -.40 | .18 | .92 | .62 |
| KISS | 420 | 10 (6, 12) | 914 | -.22 | -.26 | .14 | .91 | .64 |
| Pelotas | 424 | 13 (12, 14) | 970 | -.59 | -.43 | .32 | .88 | .52 |
| TAAG | 1,652 | 11 (10, 14) | 790 | -.41 | -.43 | .33 | .92 | .71 |
| CHAMPS-UK | 436 | 10 (5, 16) | 729 | -.65 | -.11 | .03 | .87 | .46 |
| NHANES 2003-2004 | 2,082 | 12 (6, 18) | 782 | -.49 | -.35 | .33 | .92 | .66 |
| NHANES 2005-2006 | 2,177 | 12 (6, 18) | 782 | -.54 | -.43 | .37 | .92 | .68 |
| IBDS | 622 | 13 (4, 18) | 781 | -.81 | -.53 | .47 | .91 | .61 |

CI, confidence interval; LPA, light-intensity physical activity; MPA, moderate-intensity physical activity; MVPA, moderate- and vigorous-intensity physical activity; SB, sedentary behavior; TAC, total activity counts; VPA, vigorous-intensity physical activity.

Study full names

SPEEDY: Sport, physical activity and eating behaviour: environmental determinants in young people (SPEEDY)

PEACH: Personal and Environmental Associations with Children's Health

ALSPAC: Avon Longitudinal Study of Parents and Children
CLAN: Children Living in Active Neigbourhoods

COSCIS: Copenhagen School Child Intervention Study

EYHS: European Youth Heart Study

HEAPS: Healthy Eating and Play Study

KISS: Kinder-Sportstudie

TAAG: Trial of Activity for Adolescent Girls

CHAMPS-UK: Children’s Health and Activity Monitoring for schools

NHANES: National Health and Nutrition Examination Survey

IBDS: Iowa Bone Development Study

Table S2. Correlation coefficients between accelerometer-derived metrics for the 16 studies with the walking-hour accelerometer protocol

|  | Wear time & SB | Wear time & LPA | Wear time & MPA | Wear time & VPA | Wear time & TAC |
| --- | --- | --- | --- | --- | --- |
| *r* (99% CI) | .53 (.53, .54) | .28 (.27, .29) | .03 (.02, .05) | .07 (.06, .08) | .10 (.09, .11) |
|  | SB & LPA | MVPA & SB | MVPA & LPA | MVPA & TAC | MPA & VPA |
| *r* (99% CI) | -.63 (-.63, -.62) | -.44 (-.45, -.43) | .31 (.30, .32) | .91 (.91, .91) | .58 (.58, .59) |

CI, confidence interval; LPA, light-intensity physical activity; MPA, moderate-intensity physical activity; MVPA, moderate- and vigorous-intensity physical activity; SB, sedentary behavior; TAC, total activity counts; VPA, vigorous-intensity physical activity.

**Figure**

Figure 1. The proportion of vigorous-intensity physical activity (VPA) minutes within moderate- and vigorous-intensity physical activity (MVPA) minutes

Note. Error bar indicates 99% confidence interval.
